# HoughFeature, a novel method for assessing drug effects in three-color cDNA microarray experiments

**DOI:** 10.1186/1471-2105-8-256

**Published:** 2007-07-17

**Authors:** Hongya Zhao, Hong Yan

**Affiliations:** 1 Department of Electronic Engineering, City University of Hong Kong, Kowloon, Hong Kong; 2 School of Electrical and Information Engineering, University of Sydney, NSW 2006, Australia

## Abstract

**Background:**

Three-color microarray experiments can be performed to assess drug effects on the genomic scale. The methodology may be useful in shortening the cycle, reducing the cost, and improving the efficiency in drug discovery and development compared with the commonly used dual-color technology. A visualization tool, the hexaMplot, is able to show the interrelations of gene expressions in normal-disease-drug samples in three-color microarray data. However, it is not enough to assess the complicated drug therapeutic effects based on the plot alone. It is important to explore more effective tools so that a deeper insight into gene expression patterns can be gained with three-color microarrays.

**Results:**

Based on the celebrated Hough transform, a novel algorithm, HoughFeature, is proposed to extract line features in the hexaMplot corresponding to different drug effects. Drug therapy results can then be divided into a number of levels in relation to different groups of genes. We apply the framework to experimental microarray data to assess the complex effects of Rg1 (an extract of Chinese medicine) on Hcy-related HUVECs in details. Differentially expressed genes are classified into 15 functional groups corresponding to different levels of drug effects.

**Conclusion:**

Our study shows that the HoughFeature algorithm can reveal natural cluster patterns in gene expression data of normal-disease-drug samples. It provides both qualitative and quantitative information about up- or down-regulated genes. The methodology can be employed to predict disease susceptibility in gene therapy and assess drug effects on the disease based on three-color microarray data.

## Background

Microarray experiments can produce expression data of thousands of genes simultaneously. They are useful for disease diagnosis, prognosis and treatment planning as well as the discovery and development of novel pharmaceutical products [1-11]. However, these experiments, which commonly use two colors, are costly and time-consuming, thus there is a need to improve the technology. By adding blue Alexa 594 as a dye-label, a three-color microarray experiment can be carried out. According to recent experimental and statistical analysis, there is no evidence that the inclusion of Alexa594 as the third dye-label causes additional noise or unexpected results in the data [12,13]. A three-color microarray experiment requires fewer arrays, saves samples, and simplifies the experimental process, leading to a reduction of costs and time without compromising gene expression data [12-14].

However, three-color microarrays have not been used widely yet partly because of the lack of data analysis tools. Existing data analysis methods are usually borrowed from those for dual-color microarrays and they may not be able to deal with complicated patterns in three-color data. Thus, it is important to develop new and more effective tools.

**Figure 1:**
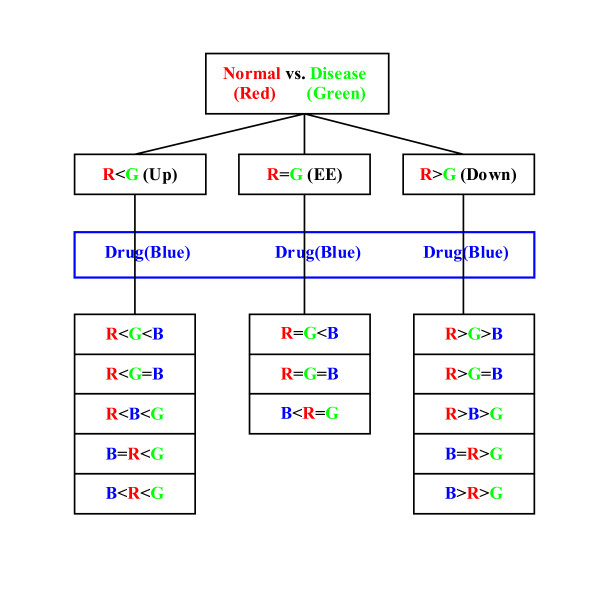
Overview of expression patterns in normal-disease-drug samples. There are 13 possible cases of gene expression patterns in three-color cDNA microarray data.

**Figure 2:**
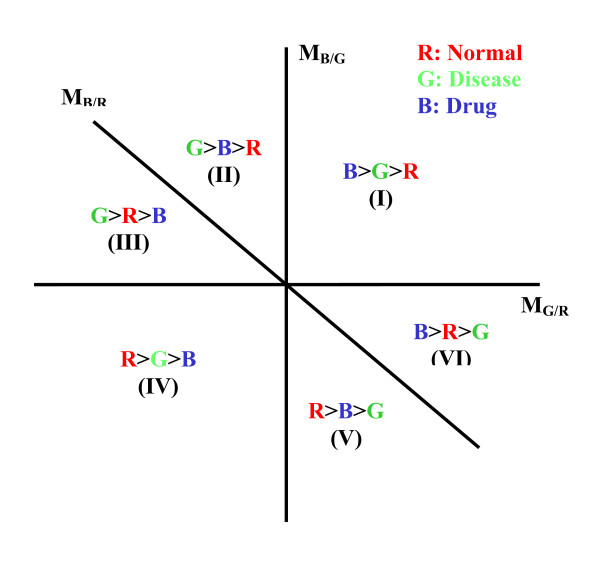
An example of the hexaMplot. Three lines divide the two-dimensional plane into six regions, corresponding to different drug effects on disease-related genes.

In [14], Zhao (the first author of this paper) et al first proposed the hexaMplot to demonstrate the interactions of gene expressions and used the method to assess drug effects based on three-color microarray data. The complicated expression patterns in three dyed samples, as demonstrated in Figure 1, can be simplified by the special coordinates of the hexaMplot. As summarized in Figure 2, the three lines, six regions, and the origin of the hexaMplot provide a lot of useful information about the alteration in the gene expression data. A more detailed description of the hexaMplot is provided in Methods.

Although the hexaMplot provides a number of useful features, it is not enough to determine drug therapeutic effects with only one plot. In [14], the correlation coefficient (CC) is used to infer the binary hypothesis of drug effects. This approach has several limitations. Firstly, statistical testing is largely dependent on the assumption of the Gaussian probability distribution, but microarray data does not follow this distribution very well [15]. So there is uncertainty in the results of the performance testing [16]. Secondly, more than two binary hypotheses (equal or not) should be used in the testing since there should be more than two cases involved in three-color microarray data [14]. Genes do not function separately but together and drugs also work on different groups of genes in different ways [17]. So it is more reasonable to divide drug effects into several levels. Finally, the testing ignores the inherent noise and variation in microarray data [18].

In order to ameliorate the previous drawbacks, a novel algorithm, HoughFeature, is developed in this paper to assess drug effects based on three-color microarray data. In the proposed algorithm, lines passing through the origin of the hexaMplot are used to characterize the drug effects on gene expressions. The direction of a line can be used to determine whether the drug has positive or negative effect on a group of genes and the line slope value can be used to measure the drug effect level. To make these assessments, a key step is to find multiple lines in the hexaMplot. The Hough transform (HT) is especially useful for this task as it has been used successfully for the detection of lines and even arbitrary curves in noisy images [19-22] and has recently been applied to microarray data biclustering [23,24]. The HoughFeature algorithm can be used to analyze gene expression data from normal-disease-drug samples systematically. Naturally, the method can be generalized to deal with more than three colors if additional colors can be used and are beneficial in microarray experiments.

In this paper, we present the methodology and application of three-color microarray data analysis to assess the complex effects of Rg1 (an extract of Chinese medicine) on Hcy (homocysteine)-treated HUVECs (Human Umbilical Vein Endothelial Cells). We classify the susceptible genes in disease and drug-treated cells into different levels according to the slopes of rays detected using our algorithm. We show that the proposed method is effective for the analysis of therapeutic and side effects of drugs on a disease. The Matlab programs of our algorithm are included in the attached files.

## Results

### The HoughFeature algorithm

In order to identify significant features in the hexaMplot, we propose the HoughFeature algorithm to assess drug effects in three-color cDNA microarray data. In our algorithm, we firstly identify differentially expressed genes under disease or drug-treated conditions. We apply the HT to the gene expression data, to detect lines passing the origin in the hexaMplot. Then the drug effects on groups of genes characterized by different lines are quantified according to their slopes. The algorithm consists of the following steps [see Additional file 1 and 2].

Input: Gene expression matrix *E*_Nxn_; Quantization step size in the parameter space *δ*; Minimum number of genes on a line *d*; and the algorithm that identifies significant genes ALG:

HoughFeature (*E*, *δ*, *d*, ALG)

Step 1: Perform ALG(E) to identify the up- and down-regulated significant genes in *E*, denoted as U and D respectively. ||U|| (||D||) denotes the number of elements in the set U (D).

Step 2: Apply the HT (see description in Methods) with quantization step size *δ *to U (D) in the hexaMplot. Corresponding to ||U|| (||D||) points in the data space, there are ||U|| (||D||) sinusoidal curves in the polar parameter space. In addition, the origin of the data space is transformed to the line *ρ *= 0 in the parameter space. Thus, we can detect lines passing through the origin in the data space by examining the Hough accumulator arrays for *ρ *= 0. We retain a line for further analysis only if it has at least *d *votes, corresponding to *d *genes, in its accumulator.

Step 3: Assuming that the detected lines are *l*_*i *_(*i *= 1, 2, ..., *p*) and their slopes are *s*_*i *_(*i *= 1, 2, ..., *p*), we can then quantify the drug effects as follows:

Case 1: *s*_*i *_< -1, the drug shows significant therapeutic effect with some positive side effects on the genes on line *l*_*i *_(in regions II and V in Figure 2);

Case 2: *s*_*i *_= -1, the drug shows significant therapeutic effect with no side effect on the genes on line *l*_*i *_(on the slant axis in Figure 2);

Case 3: -1 <*s*_*i *_< 0, the drug shows significant therapeutic effect with some negative side effect on the genes on line *l*_*i *_as (in regions III and VI);

Case 4: *s*_*i *_> 0, the drug shows significant side effect with little therapeutic effect on the genes on line *l*_*i *_(in regions I and IV).

Furthermore, the closer to -1 *s*_*i *_is, the higher the therapeutic effect with lower side effect the drug has.

Step 4: Delete the genes on the detected lines from U to update the set and repeat Step 2 until the value of every remaining accumulator is less than *d*.

### Application of the HoughFeature algorithm

A three-color cDNA microarray experiment was conducted in the Biomedical Laboratory of Hong Kong Baptist University to study the effect of Rg1 (a predominant compound of the total extract of ginsenosides in ginseng) on Hcy (homocysteine)-treated HUVECs (Human Umbilical Vein Endothelial Cells). The cDNA probes of the normal HUVECs are labeled with Cy5 (Red), these of the Hcy-related HUVECs with Cy3 (Green) and these of the Hcy-related HUVECs following treatment with Rg1 with Alex549 (Blue). Thus, there are three groups of comparisons that can be made, including normal vs. Hcy-related samples (disease group), Rg1 vs. normal (drug-related group), and Hcy vs. Rg1 group (drug-following-disease group). We can study the three groups in a single three-color microarray experiment, but would need three dual-color cDNA microarray experiments for the same task. We can see that three-color microarray experiment is indeed economical, efficient, and has potentials for other applications.

Microarray data are always subject to variations from system bias other than the biological difference between samples. So the original microarray data must be normalized to remove the systematic bias. Both experimental studies and statistical analyses have been carried out in [12,13] to verify that standard normalization methods are still applicable to three-color microarray data. In the experiment presented in this paper, we choose the non-linear Loess method [28] to normalize the data [see Additional file 3].

**Figure 3:**
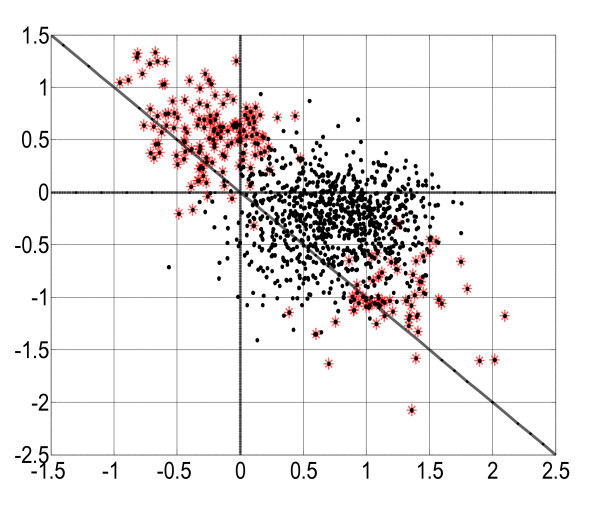
The hexaMplot of three-color cDNA microarray data from an experiment to study the disease group (Hcy) and drug-treated group (Rg1). Genes on the detected lines are shown in red. We are unable to observe line patterns in this original data space since they are embedded in noise or irrelevant data samples.

We show a hexaMplot of the normalized expression data in Figure 3. The data are distributed long along a line that slightly deviates from the slant axis. First, we apply the commonly-used SAM algorithm to identify differentially expressed genes [29]. In our analysis, we employ the paired data format of SAM, considering the expression data measured before and after the drug treatment. In Figure 3, 202 significant genes are selected with FDR = 0.05, in which 139 are up-regulated and 63 down-regulated. They are marked with red stars in Figure 3. Most of the significant genes are in the second and fourth quadrants in the hexaMplot, supporting the biological view that Rg1 has been proven to promote the angiogenesis in Hcy-related HUVECs in many medical experiments.

We analyze the effects of Rg1 based on the expression data of 202 genes and demonstrate the results in Table 1. The second column of the table shows the number of genes on each line detected in the hexaMplot. In addition to the slope (tangent value) in the third column, we also show the 95% confidence interval of the slope in the fourth column of Table 1. In the fifth column of Table 1, we define the Neg. T (negative therapeutic effect) of drug when the slope value has a negative deviation from the slant axis because the drug-related expression is opposite to the disease-related expression, that is, R is between B and G. The positive deviation can be similarly defined as Pos. T (positive therapeutic effect) because B is between R and G.

**Table 1:** Analysis of the effects of Rg1 on Hcy-treated HUVECs

Significant genes	No. of genes on each line	Tangent value	95% confidence interval	General PW-1 effect	Biological function GO term
Down-regulated (63)	7	-0.94	±0.19	Neg. T (VI)	GO: 0006468
	6	-1.05	±0.46	Pos. T(V)	GO: 0006350
	5	-1.15	±0.21	Pos. T(V)	GO: 0005515
	5	-0.67	±0.19	Neg. T(VI)	GO: 0005515
	5	-0.59	±0.19	Neg. T(VI)	GO: 0005515
	4	-0.79	±0.08	Neg. T(VI)	GO: 0007165
	4	-0.86	±0.27	Neg. T(VI)	GO: 0005515
Up-regulated (139)	12	-3.03	±0.25	Pos. T(II)	GO: 0005515
	11	-2.02	±0.18	Pos. T(II)	GO: 0005515
	11	-1.12	±0.07	Pos. T(II)	GO: 0005515
	10	-34.23	±24.60	Pos. T(II)	GO: 0016020
	9	12.35	±8.48	Side (I)	GO: 0005737
	9	-4.29	±0.90	Pos. T(II)	GO: 0005509
	9	-1.37	±0.15	Pos. T(II)	GO: 0005515
	8	-0.73	±0.11	Neg. T(III)	GO: 0005515

**Figure 4:**
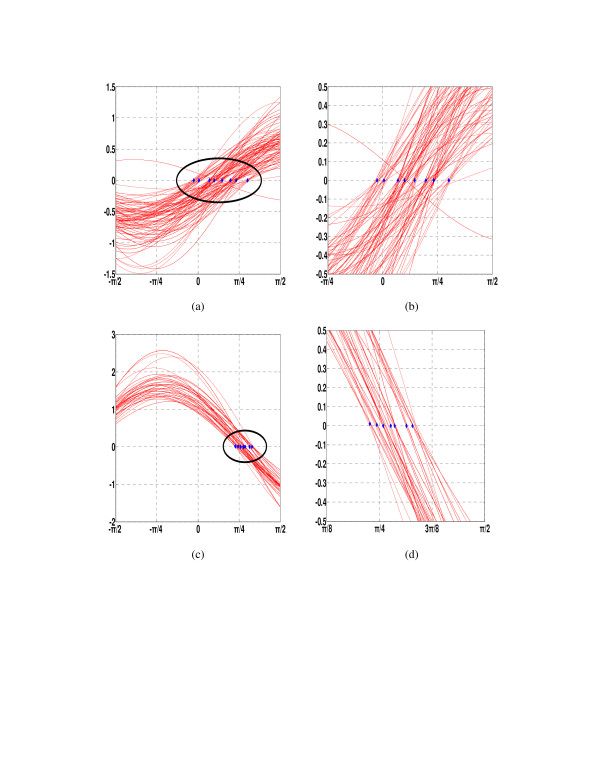
The peak points corresponding to the 15 detected lines in the polar parameter space obtained using the HoughFeature algorithm. The up-regulated genes are shown in (a) and the curve intersection regions are zoomed in and shown in (b). The down-regulated genes are shown in (c) and the curve intersection regions are zoomed in and shown in (d).

In Table 1, the genes are classified into 15 lines corresponding to different drug effect levels. We plot the gene expression data in Figure 3, where genes on the detected lines are shown in red and those not on the lines in black. In this original data space, it is not easy to observe obvious line clusters because they are embedded in noise or irrelevant data samples. However, in the Hough parameter space, each line hidden in the original data is related to a peak point, or the intersection of many sinusoidal curves. In the Hough space, we can search for the lines easily by simply filtering out the curves that do not intersect with many other curves at a common point. This is demonstrated in Figure 4, where the curve intersections correspond to distinct line clusters. Genes on the same line show similar expression patterns and similar drug effects. However, not all 202 genes are classified because some genes are located on those lines each of which has less than *d *genes and are not selected for further consideration. Due to noise and outliers, these small groups may not be meaningful statistically. This classification scheme is reasonable because genes do not function separately but together in complex biological systems, as discussed above. In many cases, it is more useful to study groups of genes together that are significantly expressed in a similar way than individual genes separately. This is why a lot of research is being carried out to analyze the functional groups of genes using the microarray technology.

We have also analyzed the biological functions of these resulting groups. Currently, the Gene Ontology (GO) is a well accepted standard for gene function categorization [30-33]. The GO project provides a controlled vocabulary for various genomic databases of diverse species in such a way that it can show the essential features shared by all the organisms. In the GO function analysis, the hypergeometric distribution is applied to calculate the probability *p *of the related GO 'biological process' terms to assess the significance of our resulting groups [31-33]. The approach is introduced in Methods. We list the most significant GO function levels of the genes on the lines detected using the HoughFeature method in the last column of Table 1.

Based on the suggestion of some biologists, we set the minimum number of genes in a functional group *d *= 7 to study up-regulated genes. The value of *d *is about 5% of significantly up-regulated genes. The genes are classified into eight lines, in which the slopes of seven lines are less than 0 in the Neg. T and Pos. T regions and one is larger than 0, to demonstrate some side effects of Rg1. Based on the GO analysis of gene functions, the Rg1 shows therapeutic effects on the Hcy-related genes in regions II and III in relation to protein binding (GO:0005515), membrane (GO:0016020) and calcium ion binding (GO: 0005509). The side effects of Rg1 are observed with the function of cytoplasm (GO: 0005737) in region I. Similarly, we select *d *= 4 in the analysis of the down-regulated genes. The genes are identified along seven lines in the Neg. T and Pos. T region. Rg1 still shows positive effects on protein binding (GO: 0005515) as well as protein amino acid phosphorylation (GO: 0006468), transcription (GO: 0006350), and signal transduction (GO: 0007165). The GO analysis shows that Rg1 indeed has therapeutic effects consistently on most of the differentially expressed genes in Hcy-related HUVECs. The drug is too powerful to make the genes regress to normal perfectly and causes some side effects. In fact, a better pattern of therapy may be obtained by adjusting the drug dosage, which always plays a key role in disease treatment.

## Discussion and conclusion

Our study shows that the HoughFeature algorithm provides a practical and effective solution to assess drug effects based on the hexaMplot for three-color cDNA microarray experiments. Although a drug has diverse actions on different genes, we can classify the effects into different levels according to the line patterns in the hexaMplot.

For multi-factorial human disease involving as many as 100 susceptible genes (e.g. heart disease, cancer, and neurodegenerative disease), the hexaMplot and HoughFeature will be useful for the analysis of gene expressions in disease and drug-treated samples. Such a framework may ultimately deliver genomic fingerprint analyses, which are predictive of disease susceptibility and drug effects, thereby permitting the implementation of disease avoidance strategies.

The drug effect assessment scheme based on our method has potential applications to drug discovery and development in both western and Chinese medicine. Western medicine has a solid theoretical foundation based on biology, biochemistry, physiology and pathology. However, it is ambiguous and difficult to explore specific activities of Chinese herbal medicine because of its complexities in components and mechanisms. Using microarray analysis as a powerful research tool, there is an opportunity to analyze genetic and biochemical data in a wide range of clinical applications [1]. Thus, there is a common platform for the research of both western and Chinese medicine, including the comparison, evaluation and integration of gene expression data.

## Methods

### Gene expression patterns in dual- and three-color microarray experiments

In general, cDNA microarray experiments using spotted arrays involve the hybridization of two differentially labeled targets on one slide. These are called dual targets or dual-dye arrays. In such an experiment, one sample is labeled with the fluorescent dye Cy5 (red) and the other is labeled with dye Cy3 (green). After image analysis and data processing, two intensities R_ij _and G_ij _(*i *= 1, ..., *n*, *j *= 1, ..., *N*) from each spot are used to denote the respective expression level of the *i*th gene under the *j*th condition. For convenience, we will omit the subscripts of R and G in the following discussions. There are two general expression patterns, equivalent (EE: G = R) and distinct (DE: G ≠ R). The relations in DE can be further divided into up-regulated (G > R) and down-regulated (G < R) expressions. Since only the difference between G and R is important, it is enough to analyze the experimental data with their ratio (G/R) or log ratio (log(G/R)). The AM plot, in which two variables AG/R=log⁡2RG
 MathType@MTEF@5@5@+=feaafiart1ev1aqatCvAUfKttLearuWrP9MDH5MBPbIqV92AaeXatLxBI9gBaebbnrfifHhDYfgasaacH8akY=wiFfYdH8Gipec8Eeeu0xXdbba9frFj0=OqFfea0dXdd9vqai=hGuQ8kuc9pgc9s8qqaq=dirpe0xb9q8qiLsFr0=vr0=vr0dc8meaabaqaciaacaGaaeqabaqabeGadaaakeaacqWGbbqqdaWgaaWcbaGaem4raCKaei4la8IaemOuaifabeaakiabg2da9iGbcYgaSjabc+gaVjabcEgaNnaaBaaaleaacqaIYaGmaeqaaOWaaOaaaeaacqWGsbGucqWGhbWraSqabaaaaa@39C3@ and *M*_*G*/*R *_= log_2_(*G*/*R*) are used, provides an effective visualization tool for dual-color microarray data [28].

Unlike the design in a dual-color microarray, a third dye-label (Alexa 594) is introduced for a third target sample in [6-9]. Previous investigations of three-color microarray technology almost all deal with the quality control or comparison of the slides [6-9]. Many designs of three-color microarray experiments, such as triple-target self, dual-target self, and single-target hybridizations, are applied to assess the slide fabrication, visualize the array morphology, and control the array quality. Research work has also been carried out to verify the fact that the inclusion of Alexa594 as a third dye-label causes no additional noise or unexpected results in the data [12,13].

In three-color microarray experiments, the normal sample is labeled with Cy5 (Red), the disease sample is labeled with Cy3 (Green), and the drug-treated sample is labeled with Alexa 594 (Blue). Three values are obtained from every spot on an array, denoted as R, G, and B respectively to represent corresponding gene expression levels. Relations among measured three-color data are naturally more complicated than those for two-color data. Figure 1 provides an overview of the 13 relations presented in [14].

### Visulization tool: the hexaMplot

In order to display gene expression patterns in three-color microarray data, the hexaMplot was first proposed in [14]. Figure 2 shows the coordinates used in the hexaMplot. The line M_G/R _= 0 (i.e. log_2_(G/R) = 0) is selected as the horizontal axis to scale the difference in the gene expression data between disease and normal cells, and the line M_B/G _= 0 (i.e. log_2_(B/G) = 0) is considered as the vertical axis to measure the difference between disease and drug-treated samples. And the line M_B/G _= -M_G/R _within the coordinates is equivalent to the equation M_B/R _= 0 (log_2_(B/R) = 0). The equation is meaningful since it can be used to measure the difference of expression patterns between drug-treated and normal samples in the absence of disease one. Therefore the line can be defined as the slant axis to measure the specific difference of gene expression. More importantly, the slant axis makes it possible to carry out drug assessment using the hexaMplot as discussed below.

In Figure 2, the three lines divide the two-dimensional plane for the hexaMplot into six regions, denoted counter-clockwise as I (B > G > R), II (G > B > R), III (G > R > B), IV (R > G > B), V (R > B > G), and VI (B > R > G), respectively. When disease genes are up-regulated (G > R), a good drug should act to decrease their expressions. This means G > B, corresponding to regions II and III. Due to the difference in drug strength or dosage, there may be three scenarios. The first case in II suggests that the drug slightly decreases the up-regulated gene expressions in disease (G > B) but these genes are still up-regulated in the disease than normal (B > R). The second case in III means that the drug significantly decreases the gene expressions to becoming down-regulated (G > R > B). The third case on the slant axis suggests that the drug effectively acts to decrease the genes expressions to the normal level (G > B = R). All three cases should be preferred because the tendency of therapy is to bring gene expression level closer to the normal level R. A similar therapeutic conclusion on the down-regulated disease genes can be drawn for regions V and VI. In contrast, side effects are produced in cases represented by region I, where the drug makes the related genes even more up-regulated after treatment than in the disease status (B > G > R), and by region IV, where the drug makes the related genes even more down-regulated after treatment than in the disease status (R > G > B).

The regions discussed above only provide a rough and qualitative assessment of drug effects. To carry out the evaluation quantitatively, we can consider lines passing through the origin in the hexaMplot. A ray in regions II, III, V or VI from the origin has negative slopes and indicates a positive drug effect. The line slope value represents the therapeutic level. When the slope has value -1 or when the line coincides with the slant axis, the gene expressions completely regress to normal without any side effect after medication. Thus, we can classify drug effects on genes into different levels based on the slopes of the rays. The assessment problem is converted to the detection of the lines passing through the origin in the hexaMplot.

### The Hough transform

The HT is the basis of the HoughFeature algorithm. The HT is widely used in image analysis and computer vision for detecting multiple lines, which may be broken and mixed with noisy and textured background [19-22]. It has also been shown recently to be an effective method for microarray data biclustering [23,24]. Many studies have been carried out on the consistency, convergence, and robustness of the HT [19-22].

The basic idea of the HT can be briefly described as follows. A line in the x-y data space is defined by

*y *= *kx *+ *b*

**Figure 5:**
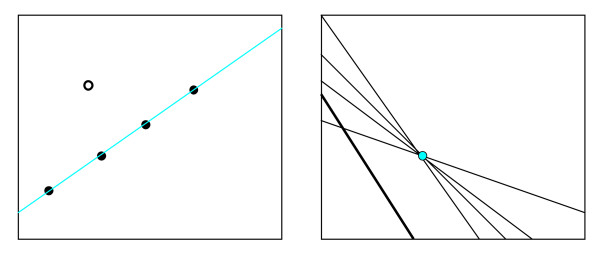
Illustration of the Hough transform in data and parameter spaces. Points in the data space (left) correspond to lines in the parameter space (right). Points on a line in the data space produce lines that intersect at the same point in the parameter space, while random points in the data space produce random lines in the parameter space.

Note that a line in the x-y space as defined by Equation (1) corresponds to a point (*k*, *b*) in the k-b parameter space. Conversely, the line in Equation (1) in the k-b space corresponds to a point (*x*, *y*) in the x-y space. If *n *points {(*x*_*i*_, *y*_*i*_): *i *= 1,...,*n*} on a line in the x-y space are known, lines obtained from all *n *points should pass through the same point in the k-b space. Figure 5 shows the relation of the lines and points in their respective spaces. Therefore, to determine lines from points, we can initialize all entries of the k-b space to zero and increase an entry by one vote when the line representing a point in the x-y space passes through it, and then find the entry in the k-b space that has the highest vote. If more than one line is to be detected, entries with local peak counts in the k-b space are located and their coordinates are used as the slopes and y-intercepts of the line. The accumulator array in the parameter space may be very large because the range of the slope is large, especially for vertical lines. Alternatively, the polar form can be used to describe a line:

*ρ *= *x *cos*θ *+ *y *sin*θ*

where *ρ *is the distance of a line to the origin and *θ *is the angle of the normal to the line with the x axis. Since *ρ *varies from -x2+y2
 MathType@MTEF@5@5@+=feaafiart1ev1aqatCvAUfKttLearuWrP9MDH5MBPbIqV92AaeXatLxBI9gBaebbnrfifHhDYfgasaacH8akY=wiFfYdH8Gipec8Eeeu0xXdbba9frFj0=OqFfea0dXdd9vqai=hGuQ8kuc9pgc9s8qqaq=dirpe0xb9q8qiLsFr0=vr0=vr0dc8meaabaqaciaacaGaaeqabaqabeGadaaakeaadaGcaaqaaiabdIha4naaCaaaleqabaGaeGOmaidaaOGaey4kaSIaemyEaK3aaWbaaSqabeaacqaIYaGmaaaabeaaaaa@32DB@ to x2+y2
 MathType@MTEF@5@5@+=feaafiart1ev1aqatCvAUfKttLearuWrP9MDH5MBPbIqV92AaeXatLxBI9gBaebbnrfifHhDYfgasaacH8akY=wiFfYdH8Gipec8Eeeu0xXdbba9frFj0=OqFfea0dXdd9vqai=hGuQ8kuc9pgc9s8qqaq=dirpe0xb9q8qiLsFr0=vr0=vr0dc8meaabaqaciaacaGaaeqabaqabeGadaaakeaadaGcaaqaaiabdIha4naaCaaaleqabaGaeGOmaidaaOGaey4kaSIaemyEaK3aaWbaaSqabeaacqaIYaGmaaaabeaaaaa@32DB@ and *θ *is limited from -*π*/2 to *π*/2, the dynamic ranges of the parameters are compressed and a small accumulator array is sufficient to find all lines. Note that if the polar equation of a line is used, each point in the x-y space corresponds to a sinusoidal curve in the ρ-θ space. Again, array entries with local peak counts should be identified and used to detect the lines [19-22].

In a hexaMplot, the lines passing through the origin are of interest and thus we are able to employ the Hough technique to detect them. In addition, the origin in the data space corresponds to the line *ρ *= 0 in the polar parameter space after the HT. As a result, the line passing through *n *points including the origin in the hexaMplot can be transformed as an accumulator array intersected by *n *- 1 sinusoidal curves and one *ρ *= 0 line. The slope of the line in the hexaMplot can be calculated based on parameters of the accumulator array and can then be used to assess the drug effect on the genes on the line [see Additional file 2].

We prefer the HT to linear regression for the following reasons. Firstly, the HT is robust to noise and outliers [19-22], and microarray data are usually very noisy. Secondly, multiple lines can be detected at the same time using the HT without knowing beforehand which points are placed on which line. We can fit a given set of points to only one line model in linear regression. Therefore, the HT is especially useful for detecting the multiple lines in the hexaMplot in microarray analysis.

In general, the HT can be used to extract multiple groups of points which form lines or curves and are corrupted by noise in a multi-dimensional space. It has been studied extensively in image processing and has many applications, including recognition of roads and buildings in digital images and videos, and biclustering of microarray data [19-27]. Biclustering has recently become a very active research topic in bioinformatics [23-27]. In biclustering, we are interested in grouping a subset of genes which have consistent expression patterns under a subset of conditions. Biclusters with constant, additive and multiplicative coherent values can all be modeled using linear equations or hyperplanes in a multi-dimensional data space. Although we do not know the gene groups and condition groups beforehand, the voting process in the parameter space reveals the consistency among the data points. That is, points forming a coherent pattern will always contribute to the same accumulator and thus produce a peak in the parameter space. The HoughFeature algorithm for three-color microarray data analysis developed in this paper can be considered as a special case of biclustering. The line patterns in the hexaMplot are multiplicative biclusters. That is, we would like to group the genes that are consistently upsor down-regulated under one condition, e.g. the disease status, compared with another condition, e.g. normal or drug-treated status.

### Hypothesis testing of the correlation coefficient

The correlation coefficient between M_G/R _= log_2_(G/R) and M_B/G _= log_2_(B/G) is defined as

γ=∑i=1N(MG/Ri−M¯G/R)(MB/Gi−M¯B/G)NSG/RSB/G,
 MathType@MTEF@5@5@+=feaafiart1ev1aqatCvAUfKttLearuWrP9MDH5MBPbIqV92AaeXatLxBI9gBaebbnrfifHhDYfgasaacH8akY=wiFfYdH8Gipec8Eeeu0xXdbba9frFj0=OqFfea0dXdd9vqai=hGuQ8kuc9pgc9s8qqaq=dirpe0xb9q8qiLsFr0=vr0=vr0dc8meaabaqaciaacaGaaeqabaqabeGadaaakeaaiiGacqWFZoWzcqGH9aqpdaWcaaqaamaaqadabaWaaeWaaeaacqWGnbqtdaqhaaWcbaGaem4raCKaei4la8IaemOuaifabaGaemyAaKgaaOGaeyOeI0Iafmyta0KbaebadaWgaaWcbaGaem4raCKaei4la8IaemOuaifabeaaaOGaayjkaiaawMcaamaabmaabaGaemyta00aa0baaSqaaiabdkeacjabc+caViabdEeahbqaaiabdMgaPbaakiabgkHiTiqbd2eanzaaraWaaSbaaSqaaiabdkeacjabc+caViabdEeahbqabaaakiaawIcacaGLPaaaaSqaaiabdMgaPjabg2da9iabigdaXaqaaiabd6eaobqdcqGHris5aaGcbaGaemOta4Kaem4uam1aaSbaaSqaaiabdEeahjabc+caViabdkfasbqabaGccqWGtbWudaWgaaWcbaGaemOqaiKaei4la8Iaem4raCeabeaaaaGccqGGSaalaaa@5A96@

where SG/R=∑i=1N(MG/Ri−M¯G/R)2N
 MathType@MTEF@5@5@+=feaafiart1ev1aqatCvAUfKttLearuWrP9MDH5MBPbIqV92AaeXatLxBI9gBaebbnrfifHhDYfgasaacH8akY=wiFfYdH8Gipec8Eeeu0xXdbba9frFj0=OqFfea0dXdd9vqai=hGuQ8kuc9pgc9s8qqaq=dirpe0xb9q8qiLsFr0=vr0=vr0dc8meaabaqaciaacaGaaeqabaqabeGadaaakeaacqWGtbWudaWgaaWcbaGaem4raCKaei4la8IaemOuaifabeaakiabg2da9maakaaabaWaaSGaaeaadaaeWaqaamaabmaabaGaemyta00aa0baaSqaaiabdEeahjabc+caViabdkfasbqaaiabdMgaPbaakiabgkHiTiqbd2eanzaaraWaaSbaaSqaaiabdEeahjabc+caViabdkfasbqabaaakiaawIcacaGLPaaadaahaaWcbeqaaiabikdaYaaaaeaacqWGPbqAcqGH9aqpcqaIXaqmaeaacqWGobGta0GaeyyeIuoaaOqaaiabd6eaobaaaSqabaaaaa@481A@ and SB/G=∑i=1N(MB/Gi−M¯B/G)2N
 MathType@MTEF@5@5@+=feaafiart1ev1aqatCvAUfKttLearuWrP9MDH5MBPbIqV92AaeXatLxBI9gBaebbnrfifHhDYfgasaacH8akY=wiFfYdH8Gipec8Eeeu0xXdbba9frFj0=OqFfea0dXdd9vqai=hGuQ8kuc9pgc9s8qqaq=dirpe0xb9q8qiLsFr0=vr0=vr0dc8meaabaqaciaacaGaaeqabaqabeGadaaakeaacqWGtbWudaWgaaWcbaGaemOqaiKaei4la8Iaem4raCeabeaakiabg2da9maakaaabaWaaSGaaeaadaaeWaqaamaabmaabaGaemyta00aa0baaSqaaiabdkeacjabc+caViabdEeahbqaaiabdMgaPbaakiabgkHiTiqbd2eanzaaraWaaSbaaSqaaiabdkeacjabc+caViabdEeahbqabaaakiaawIcacaGLPaaadaahaaWcbeqaaiabikdaYaaaaeaacqWGPbqAcqGH9aqpcqaIXaqmaeaacqWGobGta0GaeyyeIuoaaOqaaiabd6eaobaaaSqabaaaaa@47BA@. It is desirable to detect expression patterns along the slant axis, that is, a highly negative correlation is expected to show good therapeutic effect of the drug. The following hypothesis test of *ρ *is performed:

*H*_0_:*γ *≤ *γ*_0 _v.s. *H*_1_:*γ *> *γ*_0_

where *γ*_0 _is the critical value given to assess the drug effect and the critical value is subjectively set -0.8 in [14]. The drug is inferred to show significant therapeutic effect on the disease if we reject the null hypothesis *H*_0_; otherwise the drug shows little effect. As pointed out in Introduction, the methodology has several limitations.

### Function analysis of gene clusters using Gene Ontology (GO)

Functionally related genes tend to express and perform their integrated roles in modular fashions, which are often reflected by a high degree of concert of the gene reactions to stimuli such as disease or drug-related conditions [34,35]. Based on the most widely used gene functional annotation system Gene Onotolgy (GO), we apply a hypergeometric distribution to calculate the probability *p *of a GO 'biological process' category to assess the significance of a particular function group [30-33].

The annotation is quite straight forward. Given a cluster of *n *genes, we first find the set of all unique GO terms within the 'biological process' ontology that are associated with one of more of the genes of interest. Next, for each term we determine the number of the interesting genes *k *annotated at the node and the number of assayed genes *f *annotated at the node. The hypergeometric distribution is used to model the probability of observing at least *kψ *genes from the cluster of *nψ *genes by chance in a category containing *fψ *genes from all *gψ *genes spotted on the chip [31-33]. The corresponding p-value is calculated by

P(i≥k)=∑i=kf(fi)(g−fn−i)(gn),
 MathType@MTEF@5@5@+=feaafiart1ev1aqatCvAUfKttLearuWrP9MDH5MBPbIqV92AaeXatLxBI9gBaebbnrfifHhDYfgasaacH8akY=wiFfYdH8Gipec8Eeeu0xXdbba9frFj0=OqFfea0dXdd9vqai=hGuQ8kuc9pgc9s8qqaq=dirpe0xb9q8qiLsFr0=vr0=vr0dc8meaabaqaciaacaGaaeqabaqabeGadaaakeaacqWGqbaucqGGOaakcqWGPbqAcqGHLjYScqWGRbWAcqGGPaqkcqGH9aqpdaaeWaqcaawaaSWaaSaaaeaadaqadaqaauaabeqaceaaaeaacqWGMbGzaeaacqWGPbqAaaaacaGLOaGaayzkaaWaaeWaaeaafaqabeGabaaabaGaem4zaCMaeyOeI0IaemOzaygabaGaemOBa4MaeyOeI0IaemyAaKgaaaGaayjkaiaawMcaaaqaamaabmaabaqbaeqabiqaaaqaaiabdEgaNbqaaiabd6gaUbaaaiaawIcacaGLPaaaaaaabaGaemyAaKMaeyypa0Jaem4AaSgabaGaemOzayganiabggHiLdGccqGGSaalaaa@4EFC@

which is the probability of seeing something as extreme or more extreme than what was observed. Thus, the test measures whether the cluster is enriched with genes from a particular category to a greater extent than that would be expected by chance. For example, if the majority of genes in the cluster have the same biological function, then it is unlikely that this happens by chance and the category's p-value would be close to 0. When several categories' p-values are less than the threshold, it is reasonable to annotate the cluster with the category that has the smallest p-value [32,33].

## Authors' contributions

Hongya Zhao designed and implemented the algorithms, analyzed the data, and drafted the manuscript. Hong Yan conceived the project, gave suggestion to improve the algorithm, and assisted in drafting and editing the manuscript. Both authors read and approved the final manuscript.

## Supplementary Material

Additional file 1 MATLAB M-file of HoughFeature algorithm to assess drug effects based on hexaMplot. This is the main computer program of the HoughFeature algorithm. The program input is the expression data of up- or down-regulated genes and the output is the drug effect levels on different genes. Click here for file

Additional file 2 MATLAB M-file of Hough technique. This file contains a function used by the main HoughFeature program to perform the Hough transform for detecting the line patterns. Click here for file

Additional file 3 Three-color microarray experiment data. This MAT data file contains gene names and expression data, and up- and down-regulated gene indices used in the analysis described in the paper. Click here for file
